# Recombinant influenza H7 hemagglutinins induce lower neutralizing antibody titers in mice than do seasonal hemagglutinins

**DOI:** 10.1111/irv.12285

**Published:** 2014-09-12

**Authors:** Kristy Blanchfield, Ram P Kamal, Wen-Pin Tzeng, Nedzad Music, Jason R Wilson, James Stevens, Aleksander S Lipatov, Jacqueline M Katz, Ian A York

**Affiliations:** a Influenza Division, National Center for Immunization and Respiratory Diseases, Centers for Disease Control and PreventionAtlanta, GA, USA; b Carter Consulting Inc.Atlanta, GA, USA; c Battelle Memorial InstituteAtlanta, GA, USA

**Keywords:** H7 influenza viruses, hemagglutinin, immunogenicity, influenza, influenza pandemics, influenza vaccines

## Abstract

**Background:**

Vaccines against avian influenza viruses often require high hemagglutinin (HA) doses or adjuvants to achieve serological titers associated with protection against disease. In particular, viruses of the H7 subtype frequently do not induce strong antibody responses following immunization.

**Objectives:**

To evaluate whether poor immunogenicity of H7 viruses is an intrinsic property of the H7 hemagglutinin.

**Methods:**

We compared the immunogenicity, in naïve mice, of purified recombinant HA from two H7 viruses [A/Netherlands/219/2003(H7N7) and A/New York/107/2003(H7N2)] to that of HA from human pandemic [A/California/07/2009(H1N1pdm09)] and seasonal [A/Perth16/2009(H3N2)] viruses.

**Results:**

After two intramuscular injections with purified hemagglutinin, mice produced antibodies to all HAs, but the response to the human virus HAs was greater than to H7 HAs. The difference was relatively minor when measured by ELISA, greater when measured by hemagglutination inhibition assays, and more marked still by microneutralization assays. H7 HAs induced little or no neutralizing antibody response in mice at either dose tested. Antibodies induced by H7 were of significantly lower avidity than for H3 or H1N1pdm09.

**Conclusions:**

We conclude that H7 HAs may be intrinsically less immunogenic than HA from seasonal human influenza viruses.

*Please cite this paper as:* Blanchfield *et al*. (2014) Recombinant influenza H7 hemagglutinins induce lower neutralizing antibody titers in mice than do seasonal hemagglutinins. Influenza and Other Respiratory Viruses 8(6), 628–635.

## Introduction

Avian influenza viruses of H7 hemagglutinin (HA) subtype in combination with several neuraminidase (NA) subtypes pose significant pandemic risk.^1^ In poultry, these viruses are typically low pathogenicity avian influenza (LPAI) viruses causing little disease. However, spontaneous mutations can generate highly pathogenic avian influenza (HPAI) viruses.^2^ On multiple occasions, LPAI or HPAI H7 viruses have sporadically infected humans, usually causing mild disease.^1^ In two instances, H7 viruses have infected larger contingents and caused severe disease. In the Netherlands in 2003, at least 89 people were infected with an HPAI H7N7 influenza virus, including one fatality due to severe pneumonia.^3^,^4^ In China during 2013–2014, a LPAI H7N9 virus has infected several hundred humans, with many experiencing severe respiratory disease and a case fatality rate of more than>30%.^5^,^6^

Serological testing of people for evidence of infection with H7 viruses has proven difficult. Titers measured using hemagglutination inhibition (HI) and microneutralization (MN) assays have often been low or undetectable^7^–^9^ in many human cases, even with confirmed H7 virus infections.^9^–^12^ HI and MN antibodies have been readily detected in a majority of H7N9 patients, but titers are often lower than those induced by human seasonal viruses.^13^–^15^ In pre-clinical and clinical studies, vaccines against H7 viruses have typically elicited low titers or infrequent serological responses.^16^–^20^

However, the intrinsic immunogenicity of H7 HA has not been directly compared to that of human influenza viruses, which are known to induce HI antibody titers associated with protection.^21^–^23^ We therefore used recombinant HA to compare the immunogenicity of two H7 HAs with those of a seasonal H3N2 HA and a pandemic H1N1pdm09 HA in naïve mice. A/Perth/16/2009(H3N2) and A/California/7/2009(H1N1pdm09), which were components of seasonal influenza vaccines from 2010 to 2012, were chosen as representative contemporary H3 and H1 viruses. The H7 HAs were from A/Netherlands/219/2003 (NL/219) (H7N7), an HPAIV representing the Eurasian lineage of H7,^3^ and A/New York/107/2003 (NY/107) (H7N2), a LPAIV representing the North American lineage of H7.^24^

## Materials and methods

### Recombinant hemagglutinin

The purified recombinant HA and HA1 proteins were expressed and purified from baculovirus vectors in insect cells, as described for HA from A/Netherlands/219/2003(H7N7) (NL/219) (GenBank accession number AAR02640·1);^25^ A/New York/107/2003(H7N2) (NY/107) (ACC55270·1);^26^ A/California/7/2009(H1N1pdm09) (CA/07) (ACP41953·1);^27^ and A/Perth/16/2009(H3N2) (Perth/16) (ACS71642·1).^28^ In the case of H7 from NL/219, which contains a multibasic cleavage site, the HA was cleaved into the HA1 and HA2 components during purification, while the remaining three HA were all present in the HA0 form. For all HAs, a ‘foldon’ was fused to the HA to help stabilize the trimers, as described.^28^–^31^

### Viruses

Wild-type Perth/16 and 2:6 A/Puerto Rico/1/1934 (PR8) reassortants of NL/219, NY/107, and A/Texas/5/2009(H1N1pdm09) (TX05, antigenically homologous to CA/07) were used in HI and MN assays. All the viruses used in this study were propagated in 10- to 11-day-old embryonated chicken eggs and were stored as allantoic fluid. Live H7 viruses were handled in enhanced biosafety level 3 facilities.^29^

### Immunization

Groups of five female 6- to 8-week-old BALB/c mice (Jackson Laboratory, Bar Harbor, ME, USA) were immunized intramuscularly (IM), twice at 21-day intervals with either 3 or 10 μg of purified recombinant HA in sterile PBS, or with sterile PBS as a control. Blood samples were collected on days 0 (before initial immunization), 21 (prior to boost), 35, 49, and 63.

Animal experiments were conducted in animal biosafety level (ABSL) two conditions at the Centers for Disease Control and Prevention under CDC IACUC approved protocols.

### Antibody assays

All sera were treated with receptor-destroying enzyme (RDE, Denka Seiken, Tokyo, Japan) as described,^30^ resulting in a final dilution of 1:10. Post-infection ferret antisera raised against homologous viruses were used as positive controls. Naïve sera were included as negative controls in all assays and showed undetectable levels of anti-influenza antibodies.

### HI assay

HI assays for human H3N2 and H1N1pdm09 viruses were performed as described.^30^ For H7 viruses, 1·0% horse red blood cells (HRBC) were used instead of 0·5% TRBC, as recommended for avian influenza viruses.^7^ The minimum detectable limit of this assay was a titer of 10; samples with titers <10 were assigned a value of 9 for calculating geometric mean titers (GMT).

### ELISA

ELISA was performed as described.^31^ Briefly, ELISA plates were coated overnight with HA or HA1 (Influenza Reagent Resource (IRR), Manassas, VA, USA). Serial dilution of mouse sera were probed by HRP-conjugated goat anti-mouse IgG (Alpha Diagnostic, San Antonio, TX, USA). Antibody titers are given as the reciprocal of the highest dilution which gave an OD_490_ value >2 times the average of the background wells.

### Avidity assay

For avidity ELISA, serum samples were applied to wells in duplicate. After one-hour incubation, one set of samples was incubated with wash buffer and another with 4M urea (Sigma-Aldrich, St. Louis, MO, USA) for 5 min and washed twice with wash buffer, and the ELISA protocol was completed as described. The avidity index (AI) was calculated as previously described^32^ for the lowest dilution (1:2500) as AI = (U+/U-) ×100, where ‘U+’ is the OD_490_ for wells washed with urea and ‘U-‘ is the OD_490_ for wells washed with PBST.

### Microneutralization assay

MN assays were performed as described.^30^ The minimum detection limit of this assay was a titer of 20.

### Statistical analyses

Statistical analyses for serum HI, MN, and ELISA titers were performed using a linear mixed model with repeated measures, implemented in SAS, using a cutoff of *P* ≤ 0·05 for significance. Compound symmetry was used for the covariance structure, and 95% confidence intervals were also based on compound symmetry covariance to pool the variability among subgroups. For avidity ELISAs, one-way anova was performed and significance was determined using a two-sided Student's *t*-test, with a cutoff of *P* ≤ 0·05 for significance.

## Results

### ELISA responses to purified HA

**Figure 1:**
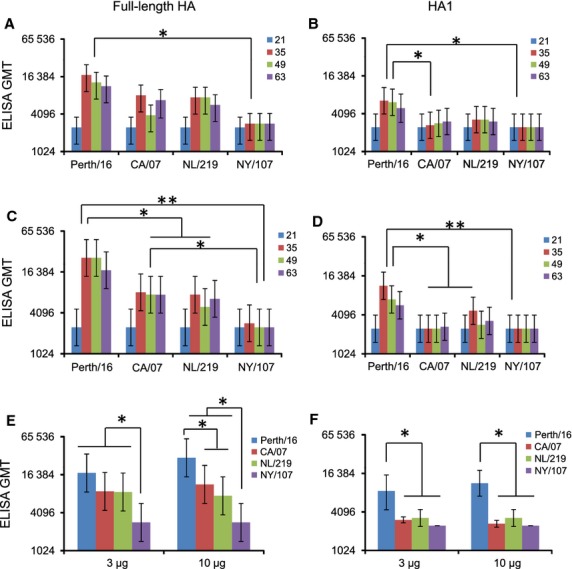
ELISA responses to recombinant hemagglutinins. Mice were immunized with (A, B) 3 μg or (C, D) 10 μg of purified recombinant hemagglutinin (HA) from Perth/16, CA/07, NL/219, or NY/107 on two occasions 21 days apart. Sera collected on days 21, 35, 49, and 63 after the initial immunization were tested by ELISA, using (A, C, E) purified recombinant homologous full-length HA or (B, D, F) HA1 as antigen. (E, F) Same experiment as A–D, showing the geometric mean titer (GMT) of the highest titer achieved by each mouse at any time point. Data are shown as GMT for each group of mice. Error bars represent 95% confidence intervals. Statistically significant differences are indicated by * (0·0001 < *P* < 0·05) or ** (*P* < 0·0001). Data are from one representative experiment of 3 replicates.

The IgG antibody response was lowest in mice immunized with NY/107 HA (Figure 1A, C), while Perth/16 HA induced the highest ELISA IgG titers (*P* < 0·05 for the 10 μg group) (Figure 1C). The NL/219 ELISA responses were similar to seasonal and pandemic virus HAs for the 3-μg dose (Figure 1A) and to H1N1pdm09 for the 10-μg dose (Figure 1C). The same pattern was seen when considering the maximum titer achieved in each animal at any time point post-immunization (Figure 1E), with Perth/16 being the highest and NY/107 being the lowest. CA/07 and NL/219 titers were similar to each other, and in the 10-μg dose, they were significantly lower than Perth/16 (Figure 1E). Thus, although by ELISA, NY/107 was less immunogenic than the other three HAs tested, NL/219 induced a similar ELISA response to the pandemic virus CA/07.

Similar results were obtained using HA1 as the antigen in ELISA (Figure 1B,D,F), although actual titers were significantly lower, as antibodies raised against the conserved stalk component of HA would not be detected. Perth/16 HA induced the greatest HA1-specific response (*P* < 0·05), while the response to CA/07 was similar to that for NL/219 or NY/107.

Antibody responses to the 3- and 10-μg doses of HA were approximately similar, suggesting that increasing the dose further would not significantly increase the antibody response.

### Hemagglutination inhibition (HI) antibodies

**Figure 2:**
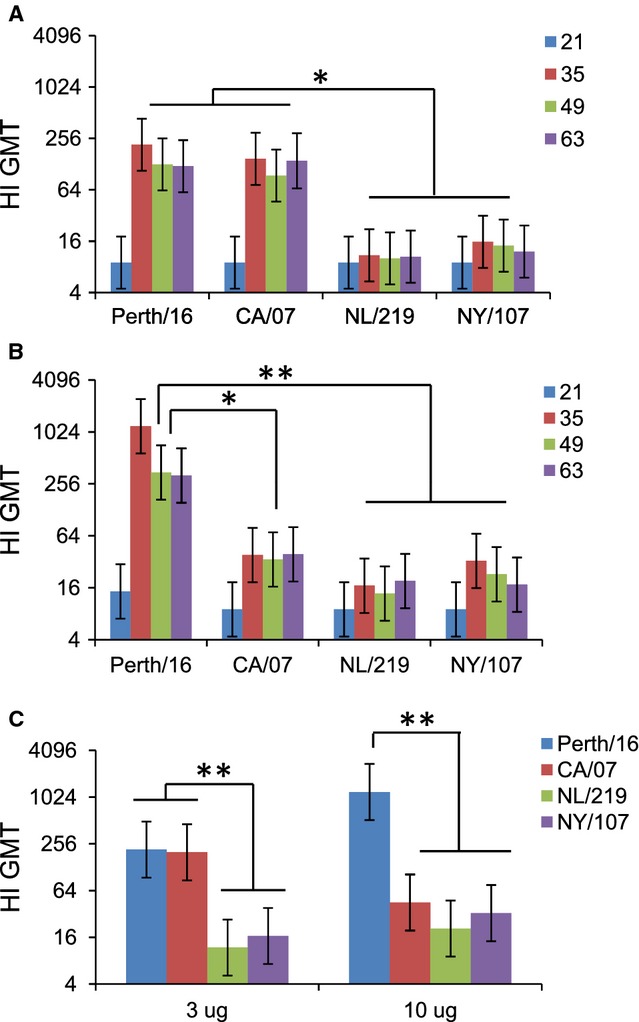
Hemagglutination inhibition (HI)responses to recombinant hemagglutinin. Mice were immunized with (A) 3 μg or (B) 10 μg of purified recombinant hemagglutinin from Perth/16, CA/07, NL/219, or NY/107 on two occasions 21 days apart. Sera collected on days 21, 35, 49, and 63 after the initial immunization were tested by HI assays (limit of detection: titer of 10), using homologous live virus as antigen. (C) Same experiment as A and B, showing the geometric mean titer (GMT) of the highest titer achieved by each mouse at any time point. Values are shown as GMT. Error bars represent 95% confidence intervals. Differences between groups are indicated by * (0·0001 < *P* < 0·05) or ** (*P* < 0·0001). Data are from one representative experiment of 3 replicates

While ELISA measures the overall antibody response to HA, the HI assay measures antibodies that bind at or near the HA receptor-binding site and inhibit virus agglutination of RBC. Sera from mice immunized with either H7 HA had significantly lower HI titers than mice immunized with either H3 or H1N1pdm09 HAs at the 3-μg dose (Figure 2A). However, at the 10-μg dose, the two H7 viruses achieved similar titers to CA/07, although all three HAs induced lower titers than Perth/16 HA (Figure 2B). A similar pattern was seen when considering only the geometric mean titers (GMT) of the maximum titers achieved in individual sera from HA-immunized mice (Figure 2C).

### Virus-neutralizing antibodies

**Figure 3:**
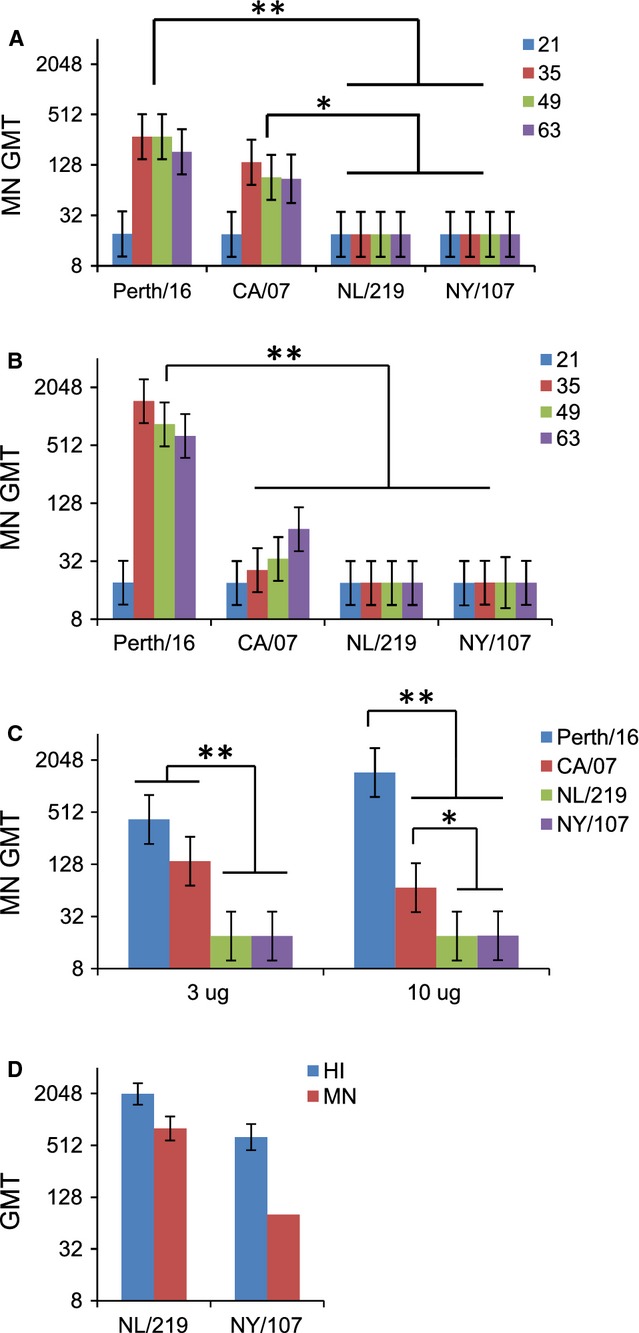
Microneutralization responses to recombinant hemagglutinin. Mice were immunized with (A) 3 μg or (B) 10 μg of purified recombinant hemagglutinin from Perth/16, CA/07, NL/219, or NY/107 on two occasions 21 days apart. On days 21, 35, 49, and 63 after the initial immunization, serum antibody titer was tested by microneutralization (MN) assays, using homologous live virus as antigen (limit of detection: titer of 20). (C) Same experiment as A and B, showing the geometric mean titer (GMT) of the highest titer achieved by each mouse at any time point. (D) MN and HI values for positive-control ferret sera, from ferrets infected with live NL/219 or NY/107. Values are shown as GMT. Error bars represent 95% confidence intervals. Differences between groups are indicated by * (0·0001 < *P* < 0·05) or ** (*P* < 0·0001). Data are from one representative experiment of 3 replicates.

Sera from mice immunized with either H3 or H1pdm HA had MN titers that were generally equal to, or higher than, corresponding HI titers (Figure 3A–C). In contrast, few animals immunized with either H7 produced detectable MN titers at any time point (Figure 3A–C). Positive-control antisera from ferrets infected with wild-type NL/219 or NY/107, which yielded HI GMT of 2031 and 640 for NL/219 and NY/107, respectively, also had MN titers (GMT of 806 and 80, respectively) that were readily detected by our MN assay (Figure 3D).

**Figure 4:**
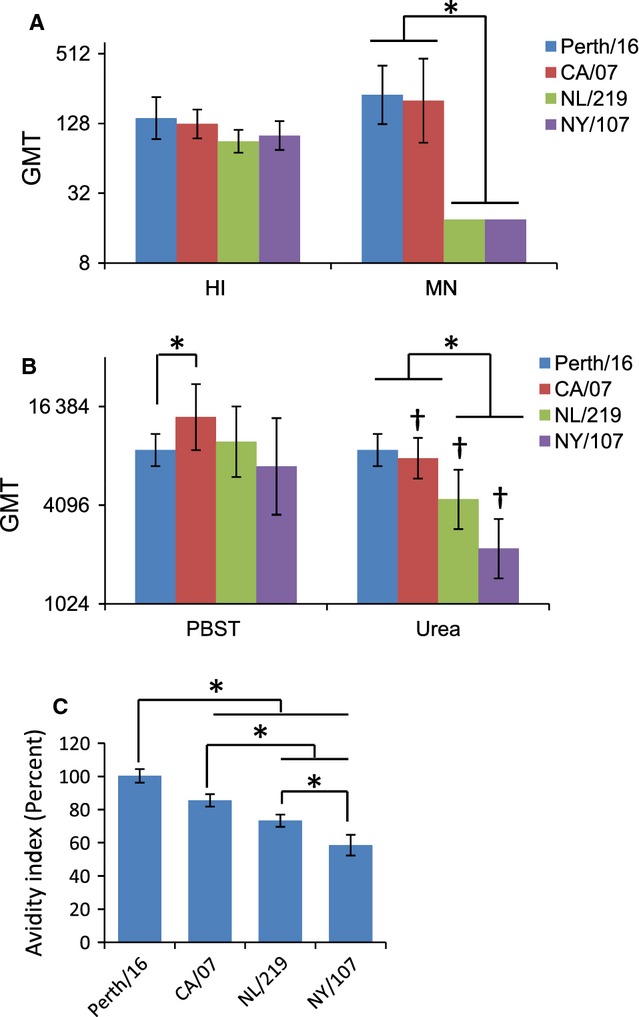
Hemagglutination inhibition (HI) and microneutralization (MN) titers and avidity ELISA for mice with HI titers between 80 and 160. Serum samples with HI titers between 80 and 160 were selected (*n* = 6 mice per group). (A) geometric mean titer (GMT) HI and MN titers for each group. (B) Avidity ELISAs were performed with the addition of 5-min incubation with either PBST or 4M urea. Values are shown as GMT. Differences between PBS and urea-treated samples are indicated by ‘†’ (*P* < 0·05). (C) The avidity index (AI) was calculated for each sample. Values are shown as average AI (percent). Error bars represent 95% confidence intervals. Differences between groups are indicated by * (0·0001 < *P* < 0·05) or ** (*P* < 0·0001).

Both HI and MN titers for H7 HAs were lower than those for H3 or H1pdm HA, but MN titers seemed relatively lower. To determine whether MN titers for the H7 HA were proportionally reduced in relation to HI titers, we identified serum samples from individuals from each group with comparable HI titers (80 or 160) and compared MN titers for each. Even when HI titers were similar, mice immunized with Perth/16 or CA/07 had significantly higher MN titers than those immunized with H7 (Figure 4A).

### Avidity ELISAs

One possible explanation for low titers detected in HI and MN assays for H7 antisera is that these assays may require higher affinity antibody binding than ELISA. To evaluate the affinity of the antibodies induced by each recombinant HA, we compared the effect of a 5-min wash in 4 m urea on ELISA titers, as previously described.^33^ Again, we used serum samples with comparable HI titers (80–160). ELISA titers for these samples were also comparable (Figure 4B, ‘PBST’), although the titer for CA/07 was slightly higher than for Perth/16 (*P *= 0·043). Urea wash (Figure 4B, ‘Urea’) did not reduce the titer in Perth/16 serum samples, but caused a significant (*P *<* *0·05) reduction in titers for CA/07, NL/219, and NY/107 sera (approximately 2-fold for CA/07 and NL/219; approximately 3·5-fold for NY/107) (Figure 4C). Following the urea wash, both NL/219 and NY/107 titers were significantly lower than either Perth/16 or CA/07 titers (*P *<* *0·05), while CA/07 and Perth/16 titers were still comparable (*P *=* *0·363). The avidity index (AI) for the H7 HAs was significantly lower than either H3 or H1pdm HA (Figure 4C) (*P *<* *0·05 versus CA/07; *P *<* *0·001 versus Perth/16).

## Discussion

Human serological responses to H7 viruses appear to be weaker than those to seasonal influenza strains.^16^–^20^ In particular, even people known to have been infected with H7 subtype influenza viruses rarely develop high titers of antibodies, as measured by HI or MN assays.^9^,^10^,^34^,^35^ While this may reflect the often-superficial nature of infection with H7 viruses, which have until recently often caused localized conjunctivitis rather than respiratory or systemic disease, this was also seen with people immunized with H7 vaccines,^16^–^18^ suggesting that humans may generally respond poorly to H7 subtypes of HA. However, in the absence of studies directly comparing the H7 antibody response to H1N1pdm09 and H3N2 strains, it has not been clear that H7 is exceptional. Furthermore, it is not clear whether any difference in response might be related to intrinsic aspects of the HA, to pre-existing immunity in humans, or to some aspect of the virus or vaccine. To distinguish between these possibilities, we immunized naïve mice with purified recombinant HA from two human viruses, H1N1pdm09 and H3N2, and two H7 viruses and compared the antibody responses.

All four HA were roughly similar in their ability to induce IgG that bound to HA (as measured by ELISA), although the H7s induced slightly lower responses than seasonal HA. When measured by HI, however, both H7 HAs were considerably less immunogenic than either H3 or H1pdm HAs. HI assays measure the ability of antibodies to prevent RBC hemagglutination by influenza viruses, mainly due to high-affinity binding near the receptor-binding site (RBS),^36^ while ELISA can also detect lower-affinity binding to any part of the HA. Our results here are consistent with clinical findings that HI titers following infection with H7 influenza viruses or immunization with inactivated H7 vaccines tend to be low.^9^–^12^,^16^–^20^

The microneutralization (MN) assay is another widely used assay for measuring antibody responses to influenza viruses. MN assays detect antibodies that block virus infection and/or replication and thus are relevant for protection against infection. By this assay, the difference between responses to HA from H7 and from human viruses was most obvious. Even when limiting analysis to sera from H7-immunized mice showing clearly detectable HI responses, MN responses were absent or very low (Figure 4A). In contrast, all mice immunized with H3 HA, and almost all those immunized with H1pdm HA, developed strong MN titers. Again, these results are consistent with observations in humans, in which H7 vaccinees had low neutralizing antibody titers despite easily detectable ELISA antibody responses.^37^

Thus, the antibody response to H7 HAs showed discordant results depending on the assay used. If antibodies are being generated to H7 viruses (as shown by ELISA), why are they poorly detected in HI and MN assays? One possible explanation is that the assays used are not capable of properly detecting H7-specific antibodies. The fact that both HI and MN assays, which measure antibodies in very different ways, show very low titers to H7, while positive-control sera gave clear reactions in each, argues against this possibility. The low MN titers for the H7 viruses raise the possibility that these viruses may have some intrinsic resistance to antibody-mediated neutralization. The fact the HI titers were also lower for H7 viruses makes this somewhat less likely, although it remains possible that some aspect of the receptor-binding site in these H7 viruses makes it resistant to antibody blockade. Other possibilities are that H7 antibodies target non-neutralizing regions of HA or that they bind with such low affinity that they are unable to block infection.

To test whether the antibody response to H7s disproportionately targets the stalk region of HA,^37^ we used HA1 as the antigen in ELISAs. While titers against HA1 were low compared to those against the full-length HA, NL/219 and the seasonal HAs showed a similar reduction in titer of about 40–50% (Figure 1D). However, as the HA1-specific responses for NL/219 and NY/107 were similar to that for CA/07 (*P* > 0·05), our data do not support the explanation that the H7 and H1pdm HAs induced different levels of HA1-specific antibodies.

Antibodies produced by B cells normally increase in affinity through the course of an infection, as activated B cells undergo somatic hypermutation of their immunoglobulin variable regions.^38^ Avidity ELISA showed that NY/107 did indeed induce low-affinity antibodies, as a brief urea wash reduced the bound antibody titer significantly (Figure 4B,C). Antibodies induced by NL/219 were also of relatively low affinity, although only modestly (but significantly) lower than those induced by CA/07. Not only did Perth/16 HA induce the highest titers of antibodies as measured in all assays, the affinity of these antibodies was also high, with titers being unaffected by the urea wash. Thus, the most likely explanation for the difference in HI and MN titers induced by H7 compared with seasonal and pandemic HA is that the former induce predominately low-affinity antibodies.

The molecular mechanism(s) for the difference in immunogenicity is unknown. As in these experiments (unlike in humans), mice had no prior exposure to seasonal HA, previous priming is not a critical factor. In addition, as purified recombinant HA was used as an immunogen, viral, or vaccine factors other than HA would not affect the response. This suggests that intrinsic features of the HA itself may affect immunogenicity, perhaps through differential induction of inflammatory responses.

It is important to note that our results are based on immunization with purified recombinant protein, demonstrating that immunogenicity is intrinsic to the protein itself. Purified proteins are intrinsically less immunogenic than the same protein in standard inactivated split vaccines, which provide adjuvant factors such as viral nucleic acids.^39^ Similarly, viral infection provides strong inflammatory signals that might be able to overcome low intrinsic immunogenicity. For example, even though immunization of mice with recombinant H7 from NL/219 and NY/107 induced low or no MN titers, positive-control serum from ferrets infected with these viruses had readily detectable MN antibodies (Figure 3D). Similarly, H7N9 human infections, most of which caused severe disease with inflammation,^40^ often lead to detectable if relatively low serological responses.^13^–^15^

In humans infected with avian influenza, weak immunogenicity may also reflect the lack of prior exposure to avian HA, compared with the repeated exposure to seasonal virus HA that may prime for subsequent responses. Similarly, vaccine or virus features other than HA may influence immunogenicity. Nevertheless, our findings suggest that, all other factors being equal, NL219 and NY107 HAs may be less immunogenic than seasonal H3 or H1pdm HA. In particular, the low level of H7-neutralizing antibody responses observed in this study suggests that H7 vaccines may require strong adjuvants or other strategies to induce antibody titers associated with protection in a majority of vaccinees.
